# Sensitive and Enzyme-Free *Pseudomonas aeruginosa* Detection and Isolation via DNAzyme Cascade Triggered DNA Tweezer

**DOI:** 10.4014/jmb.2407.07006

**Published:** 2024-08-09

**Authors:** Furong Liu, Jingyuan Xu, Lihua Yang

**Affiliations:** 1 Department of Urology, People’s Hospital of Chongqing Liang Jiang New Area, Chongqing, 401147, P.R. China; 2 Medical insurance pricing department, People’s Hospital Of Chongqing Liang Jiang New Area, Chongqing, 401147, P.R. China

**Keywords:** *Pseudomonas aeruginosa*, DNAzyme, DNA tweezer, urinary tract infections, magnetic nanoparticles

## Abstract

Effective isolation and sensitive detection of *Pseudomonas aeruginosa* (*P. aeruginosa*) is crucial for the early diagnosis and prognosis of various diseases, such as urinary tract infections. However, efficient isolation and simultaneous detection of *P. aeruginosa* remains a huge challenge. Herein, we depict a novel fluorescence assay for sensitive, enzyme-free detection of *P. aeruginosa* by integrating DNAzyme cascade-induced DNA tweezers and magnetic nanoparticles (MNPs)-based separation. The capture probe@MNPs is capable of accurately identifying target bacteria and transporting the bacteria signal to nucleic acid signals. Based on the DNAzyme cascade-induced DNA tweezers, the nucleic acid signals are extensively amplified, endowing the method with a high sensitivity and a low detection limit of 1 cfu/mL. In addition, the method also exhibits a wide detection of six orders of magnitudes. The proposed method could be extended to other bacteria detection by simply changing the aptamer sequence. Taking the merit of the high sensitivity, greatly minimized detection time (less than 1.5 h), enzyme-free characteristics, and stability, the proposed method could be potentially applied to diagnosing and preventing diseases caused by pathogenic bacteria.

## Introduction

Urinary tract infections (UTIs) are prevalent bacterial illnesses commonly acquired in communities and hospitals [1, 2]. *Pseudomonas aeruginosa* (*P. aeruginosa*) is one of the most common nosocomial infections associated with poor prognoses [3, 4]. *P. aeruginosa* can induce acute necrotizing pneumonia and urinary tract infections [5]. Several techniques have been devised to identify *P. aeruginosa*, including PCR-based approaches [6, 7], immunological methods [8], and biosensors [9]. Fluorescent biosensors have proven practical and potentially feasible in detecting *P. aeruginosa* among various approaches [10, 11]. However, the sample matrices can easily interfere with the fluorescent signals, restricting the practical use of fluorescence biosensors in actual samples.

The magnetic nanoparticles (MNPs)-based separation technique has garnered significant interest in biological analysis as a sample preparation method due to the easily manipulable adsorption properties of MNPs, which may be controlled using a magnet [12, 13]. Thus, various functional MNPs-based separation techniques have been employed as sample preparation methods to effectively enrich targets and eliminate interfering substances in complex samples, thereby significantly improving the anti-interference capability [14, 15]. However, the MNPs-based methods are insufficiently sensitive to detect low concentrations of pathogenic bacteria. Recently, a diverse array of MNPs-based methods has been proposed by integrating with isothermal signal amplification strategies, including polymerase chain reaction (PCR) [16], rolling circle amplification (RCA) [17], and recombinase polymerase amplification [18]. For example, sensitive bacteria detection methods have been proposed by integrating MNPs-based genomic DNA isolation and PCR for signal amplification. However, the signal amplification by these isothermal signal amplification strategies requires multiple enzymes and rigorous experimental conditions. In particular, the RCA-based methods necessitate the T4 DNA ligase and DNA polymerase, whereas the PCR-based methods necessitate the polymerase and thermal cycle.

DNAzyme is a catalytic nucleic acid capable of catalyzing various chemical processes [19]. Due to its superior stability and easy-to-produce capability, DNAzyme has been extensively used in biosensing, logical computing, and nanomachine engineering [20, 21]. However, few reports exist on constructing a DNAzyme cascade for *P. aeruginosa* detection. Furthermore, the sensitivity of DNAzyme cascade-based signal amplification techniques needs further enhancement.

DNA tweezers are nano-devices constructed by Watson-Crick base pairing among several DNA sequences. They possess several distinct advantages, such as a predictable structure, controllable construction, excellent biocompatibility, and easy modification. DNA tweezers have been extensively utilized in biosensing, logic processing, and medication delivery [22]. The DNA tweezers can transition between open and closed states in response to many external stimuli, including nucleic acids, proteins, enzymes, metal ions, and pH levels [23, 24].

This study proposes an innovative technique for rapid, sensitive, enzyme-free, and accurate detection of *P. aeruginosa* by integrating DNAzyme cascade-induced DNA tweezers and magnetic separation. Initially, the capture probe composing an aptamer sequence and a complementary DNA (cDNA) is loaded on the surface of MNPs, with the aptamer acting as the recognition component for *P. aeruginosa*. Upon identification of *P. aeruginosa*, the cDNA sequence was released to initiate the DNAzyme cascade, which activated a DNA tweezer-based signal reaction. Due to its elegant design, this approach can achieve the selective enrichment and fluorescence determination of bacteria in an enzyme-free way. The method’s applicability was assessed by detecting *P. aeruginosa* in artificially created clinical samples, demonstrating its potential for early detection and prevention of infections in nursing.

## Experimental Section

### Materials and Chemicals

The oligonucleotides (Table S1) utilized in this study were procured from Sangon Biotechnology Co., Ltd.(China). Phosphate buffer saline (PBS) and sodium chloride were purchased from Shanghai Sangon Biotechnology Co., Ltd. (China).

### Preparation of the Capture probe@MNPs

The carboxyl-modified MNPs were initially synthesized using a solvothermal reduction method. The aptamer@MNPs were subsequently prepared. The carboxyl-modified MNPs (15 mg) were dispersed using sonication in 500 μl of Tris-HCl (20 mM, pH 5.5). Next, 250 μl of EDC (50 mM) and 250 μl of NHS (50 mM) were added to the suspension and heated at 37°C with continuous stirring for 30 min to activate the carboxyl groups of MNPs. Subsequently, the suspension was incubated at 3°C for 30 min with 0.8 nM of amino-modified aptamer. The unfixed moieties were subsequently removed through magnetic separation. The resultants were washed three times with Tris-HCl buffer (20 mM, pH 7.4). Lastly, the aptamer-MNPs were blocked by 1 mg/ml BSA in Tris-HCl buffer (20 mM, pH 7.4). The aptamer@MNPs were resuspended in 1 ml of Tris-HCl buffer (25 mM, pH 8.5) containing 0.3 M NaCl. Subsequently, the suspension was incubated at 37°C in the dark for 40 min, and 0.8 nM of cDNA was added. Lastly, the MNPs modified by the hybrid of aptamer and cDNA (aptamer/cDNA@MNPs) were washed with Tris-HCl buffer (20 mM, pH 7.4) to remove the unfixed cDNA. The fluorescent probe@MNPs was employed to detect *P. aeruginosa*. The MNPs were stored at 4°C until use.

### Detection Procedures

The capture probe@MNPs were suspended in 50 μl of Tris-HCl buffer (20 mM, pH 7.4). Next, 30 μl of *P. aeruginosa*-containing solution was added to the suspension. The mixture was incubated on an agitator at 37°C in the dark for 40 min. After magnetic separation, 10 μl of the S probe, 10 μl of the DNA tweezers, and 5 μl of the L probe were added to the supernatant. The fluorescence spectra of the supernatant were determined by collecting it into a cell after incubation for 40 min. The excitation/emission wavelengths of 494/525 nm were used to measure the fluorescence intensities.

## Results and Discussion

### The Working Principle of the Proposed Method for *P. aeruginosa* Detection

**Fig. 1:**
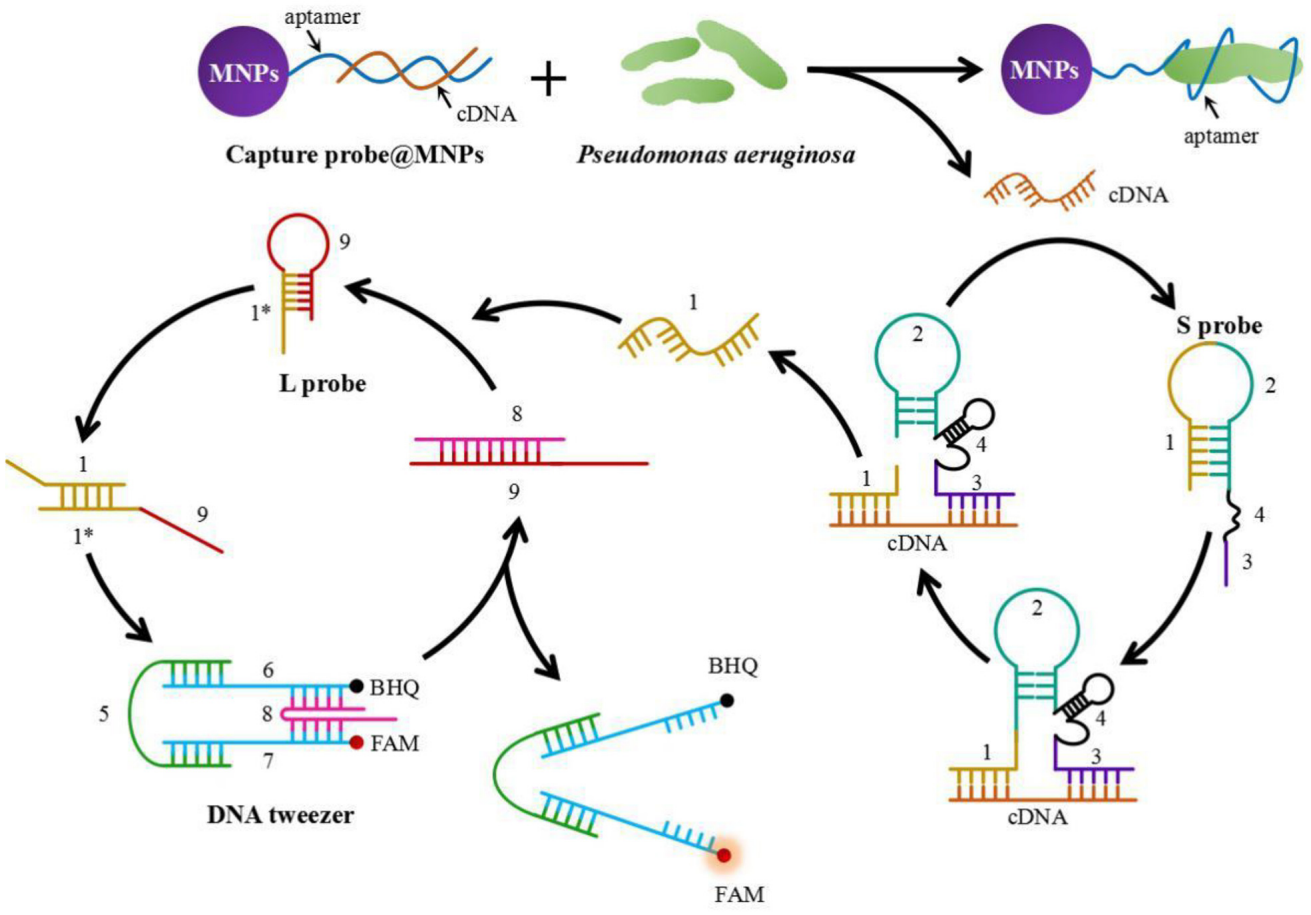
The working mechanism of the method for sensitive and enzyme-free bacteria detection.

Fig. 1 illustrates the schematic illustration of a DNA tweezer dependent on aptamer-modified MNPs for the sensitive and enzyme-free detection of *P. aeruginosa*. The aptamer was used as the biorecognition element for *P. aeruginosa* to prepare the capture probe-modified MNPs (capture probe@MNPs). In this research, the F23 aptamer, which has a high affinity and specificity to *P. aeruginosa* and has been widely applied for the construction of aptamer-based *P. aeruginosa* detection platforms, was used. The F23 aptamer was discovered using an *in vitro* evolution process called systemic evolution of ligands by exponential enrichment (SELEX), and various *P. aeruginosa* detection methods have been constructed based on the selected F23 aptamer. The cDNA was hybridized with the aptamer of the functionalized MNPs to initiate a DNAzyme cascade using DNA tweezers. In the presence of *P. aeruginosa*, the aptamer preferentially binds to the bacteria, releasing cDNA from the MNPs. Intramolecular hybridization initially suppresses the catalytic activity of “2” in the DNAzyme cascade. The released cDNA will hybridize with “1” and “3” in the presence of cDNA, producing an active DNAzyme. This enzyme subsequently cleaves the self-strand at the specific site with the help of Mg^2+^. The cleaved DNA fragments (“1”) are separated from the DNAzyme due to their low affinity with the cDNA. Meanwhile, the cDNA is released and binds to an additional S probe, initiating a new activation cycle. Consequently, the “1” sequence can anneal with the “1*” section of the L probe, thereby revealing the “9” toehold. The “9” toehold bonds with the linker (sequences “8”) of DNA tweezers through a strand displacement reaction, resulting in the opening of the DNA tweezers. Meanwhile, the “1” sequence was released by the formation of a duplex by “8” and “9”, which subsequently circularly opened the next DNA tweezers, resulting in the amplification of fluorescent signals.

### Feasibility Analysis

**Fig. 2:**
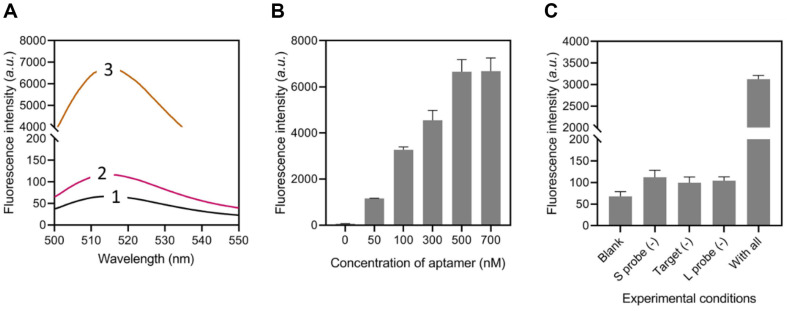
Feasibility analysis of the proposed method. (**A**) Fluorescence spectrum of the FAM-labeled cDNA during the construction of the capture probe@MNPs. Line 1: MNPs; Line 2: MNPs + cDNA; Line 3: MNPs + cDNA + aptamer. (**B**) Fluorescence intensity of the capture probe@MNPs with different concentrations of aptamer. (**C**) Fluorescence intensity of the method when essential components existed or not.

To confirm the fixation of the modified hybrid of aptamer and cDNA on the surface of MNPs, the end of the cDNA was tagged with carboxyfluorescein (FAM). The fluorescence intensity of MNPs, FAM-labeled cDNA (FAM-cDNA)/MNPs, and capture probe@MNPs were quantified in Tris-HCl solutions with a concentration of 20 mM. The MNPs were separated via magnetic separation, and the fluorescence intensities were evaluated following incubation with either aptamer or cDNA. As depicted in Fig. 2A, the fluorescence signal of MNPs is negative. Moreover, the fluorescence intensity of the capture probe@MNPs was significantly elevated, suggesting the successful immobilization of the aptamer/cDNA duplex (capture probe) onto the MNPs’ surface. In contrast, the FAM-cDNA/MNPs exhibited a minimal increase in fluorescence intensity, indicating that the aptamer plays a vital role in forming the capture probe@MNPs complex. The result in Fig. 2B showed the fluorescence intensity exhibits a positive correlation with the concentration of aptamers, demonstrating that aptamer mediated the fixation of cDNA on MNPs.

Several samples with varying settings were created to examine the viability of the DNAzyme cascade-dependent DNA tweezer. Fig. 2C demonstrates that the blank sample exhibits low fluorescence intensity because the DNA tweezer is in a “close” state (sample 1). Sample 2, in the absence of the S probe, has a feeble fluorescence intensity, suggesting no occurrence of DNA cascade. This indicates that the DNA tweezer remains closed. Sample 3 also exhibits a low fluorescence intensity due to the lack of target bacteria. The sample 4 exhibits a relatively low fluorescent signal, excluding the L probe. The “8” sequence was liberated upon the inclusion of the L probe, leading to the activation of DNA tweezers and a significant increase in fluorescence intensity.

### Parameters Optimization of DNAzyme Cascade-Dependent DNA Tweezers

**Fig. 3:**
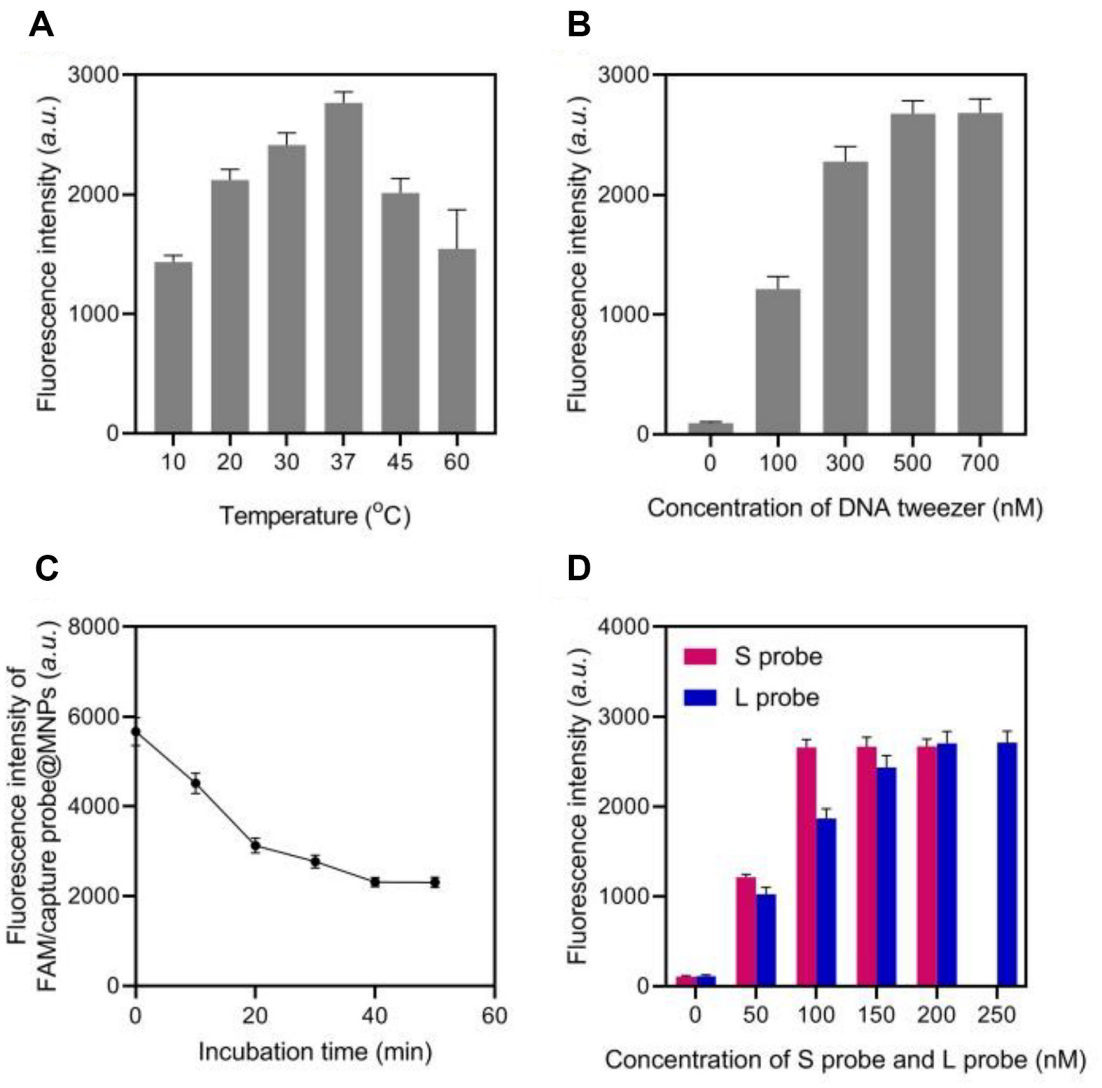
Optimization of experimental parameters. Fluorescence intensities of the method with different experimental temperatures (**A**) DNA tweezer concentrations (**B**) incubation time (**C**) and concentration of S probe and L probe (**D**).

The incubation temperature is a crucial factor that can affect the thermal stability of DNA tweezers and the hybridization efficiency between target DNA and capture DNA. Therefore, the reaction temperature was first optimized in this study. The fluorescent signal initially increases with the incubation temperature. Subsequently, it significantly reduces when the temperature exceeds 37°C (Fig. 3A). This phenomenon can be explained by the reduced effectiveness of DNA binding between the target and capture DNA at elevated reaction temperatures. Therefore, the incubation temperature was selected to be 37°C.

The concentration of DNA tweezers is the primary factor determining their fluorescent intensity. The concentration of DNA forceps has been optimized from 100 nM to 700 nM. The concentration of DNA tweezers significantly increases the fluorescent intensity. Additionally, the fluorescent signal remains consistent when the concentration of DNA tweezers is approximately 500 nM, suggesting that the DNA tweezers are nearly saturated at this concentration (Fig. 3B).

The temperature was maintained at 37°C during the investigation of the incubation time of capture probe@MNPs with bacteria. The results indicate that the fluorescence intensity increases as the incubation time increases from 0 to 40 min. However, the fluorescence intensity reaches a plateau after 40 min. This implies that the competitive substitution of cDNA by bacteria can be completed in 40 min. Consequently, the linearity of the procedure for bacteria is investigated for 40 min (Fig. 3C). The optimized concentrations of the S and L probes were 100 nM and 200 nM, respectively, as demonstrated in Fig. 3D.

### Sensitivity and Specificity of the Method

**Fig. 4:**
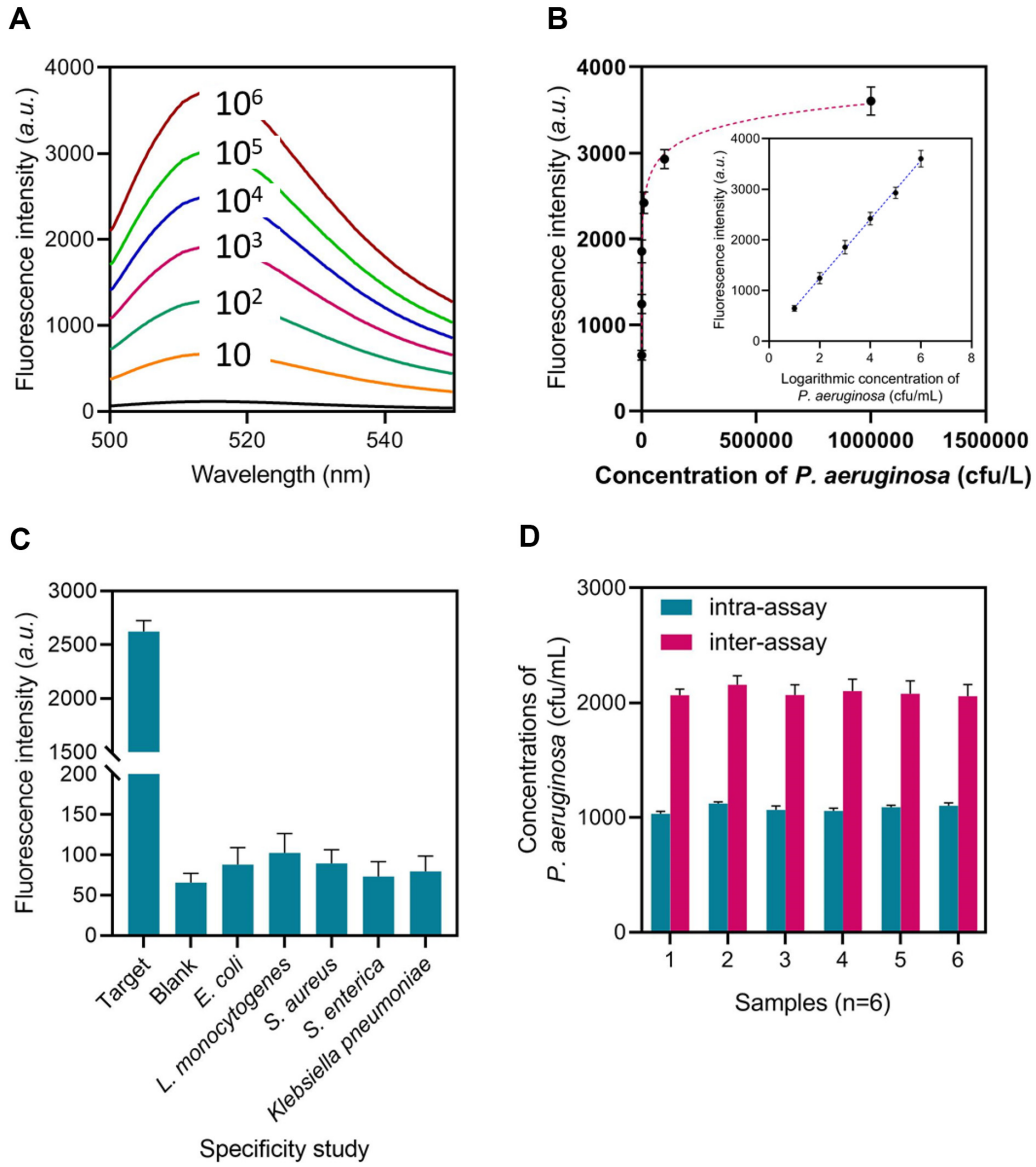
Analytical performance of the method. (**A**) The method’s fluorescence spectrum when detecting different concentrations of *P. aeruginosa*. (**B**) The correlation between the peak fluorescence intensity and the concentration of *P. aeruginosa*. (**C**) The method’s fluorescence intensity when detecting different bacteria. (**D**) The calculated *P. aeruginosa* concentration with the same batch and distinct batches of the method.

The fluorescence intensity of the DNA tweezers in the supernatant was measured for quantitative analysis of *P. aeruginosa*. Fig. 4A demonstrates that the fluorescence intensity peaked when the concentration of *P. aeruginosa* grew from 10 CFU/ml to 10^6^ CFU/ml. Fig. 4B illustrates a clear proportionality between the fluorescence intensity and the logarithm of *P. aeruginosa* concentration. The calibration curve as an inset in Fig. 4B, demonstrates a linear regression equation of F = 582.6*lgC + 77.88 with a determination coefficient of R^2^ = 0.9475. In this equation, F represents the fluorescence intensity, while C (CFU/ml) represents the concentration of *P. aeruginosa*. Furthermore, according to the 3δ rule, the limit of detection (LOD) is determined to be 1 CFU/ml (LOD = 3SD/slope, *n* = 10).

This method was employed to detect *P. aeruginosa* (10^4^ CFU/ml) and four other pathogens (*E. coli*, *L. monocytogenes*, *S. aureus*, *S. enterica*, *Klebsiella pneumoniae*) with the same concentrations to evaluate the specificity. The same conditions were used to incubate these samples with capture@MNPs. *P. aeruginosa* exhibits an apparent increase in fluorescence signal, as illustrated in Figure 4C. Meanwhile, the fluorescence signals of the four negative controls are less than 10% of those of *P. aeruginosa*. The results indicate that the aptamer-based method has a high level of specificity and selectivity for *P. aeruginosa*.

The method’s reliability was evaluated by measuring the parallel samples with the same batch and distinct batches. The method’s reproducibility is demonstrated by the fact that the intra-assay (same batch) and inter-assay (different batches) relative standard deviations (RSDs, *n* = 6) are all less than 5.4% (Fig. 4D).

### Detection of *P. aeruginosa* in Constructed Human Serum Sample

**Fig. 5:**
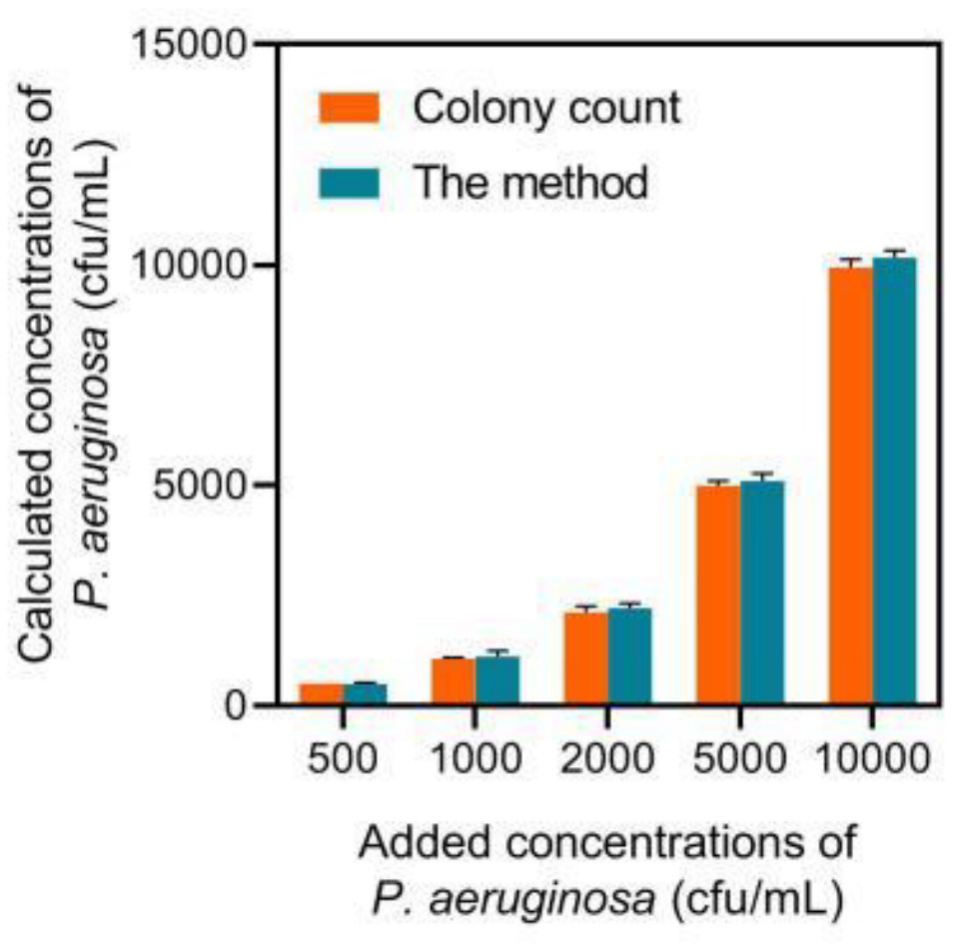
Calculated concentration of *P. aeruginosa* by the method and the colony count method.

The performance of this DNA tweezer, which relies on MNPs, was assessed by detecting *P. aeruginosa* in an artificially created human serum sample. The spiked samples exhibited recovery rates ranging from 95.0% to 107.4%, whereas the relative standard deviations (RSDs) of six repeated assessments varied from 5.32% to 8.51%(Fig. 5). These results illustrate the practicality and suitability of this approach for detecting *P. aeruginosa* in human serum.

## Conclusion

Concisely, we have created a highly responsive and enzyme-independent method for detecting *P. aeruginosa*. This involves combining the DNAzyme cascade-induced DNA tweezer with magnetic separation. Our method has several confirmed advantages: (1) It can efficiently capture and quantify *P. aeruginosa* using magnetic and fluorescence techniques in less than 1.5 h. (2) The method does not require complex equipment or sample preparation. (3) The DNAzyme cascade assay amplifies the signal for highly sensitive target detection. (4) Furthermore, the method can be used not only for detecting *P. aeruginosa*, but also as a versatile platform for detecting various targets by simply changing the corresponding aptamer and cDNA. As a potential application, it offers a novel approach to tracking low levels of bacteria in clinical samples, providing a vital tool for fundamental biomedical research and clinical diagnosis. However, the preparation of the capture probe@MNPs was somewhat complicated which may limit its application in resource-limited settings. In the future, we will integrate the whole sensing system into paper or microfluidics to establish a portable and resource-friendly kit.

## Supplemental Materials

Supplementary data for this paper are available on-line only at http://jmb.or.kr.
