# Supplementary material for: Sensitive and Enzyme-Free Pseudomonas aeruginosa Detection and Isolation via DNAzyme Cascade Triggered DNA Tweezer
Source: J Microbiol Biotechnol. 2024 Aug 9;34(9):1919–25. doi: 10.4014/jmb.2407.07006 (PMC11473567; doi:10.4014/jmb.2407.07006)
Supplement: Supplementary file 1 [file jmb-34-9-1919-supple.pdf]

J. Microbiol. Biotechnol. 2024. 34(9): 1919-1925
https://doi.org/10.4014/jmb.2407.07006

Supplementary Table

Table S1. The oligonucleotides used in this research.

Title	Sequences (5’ to 3’)
F23 aptamer	CCC CCG TTG CTT TCG CTT TTC CTT TCG CTT TTG TTC GTT TCG TCC CTG CTT CCT TTC TTG-C6-NH2
cDNA	AC GAA CAA AAG CGA
S probe	
6	GTT GGA GCG ACA TTA GAG AGC TAC AA-FAM
7	DABCYL-GTA GCC TCC TGT CCT ATC TAT GAT GG
5	CTA ATG TCG CTC CAA CAA CCA TCA TAG ATA GGA C
8	TTG TAG CAC AGG CTA CCG

Supplemented experimental section

Preparation of P. aeruginosa samples

The bacteria strain *P. aeruginosa* (ATCC15442) was inoculated and cultivated in Luria-Bertani broth at 37°C overnight. The mixture was mixed at 200 rpm using a ZHWY-103B shaker. The bacterial cells were isolated from the bouillon after being centrifuged at 10000 rpm (9167×g) for 1 min before use. Subsequently, the isolated bacterial cells were washed twice with Tris-HCl buffer (20 mM, pH 7.4). The optical density at 600 nm (OD600) (optical path length 10 mm) was measured to achieve the desirable concentration of bacteria. Following this, 10-fold dilutions were prepared in Tris-HCl (20 mM, pH 7.4) and plated out on Luria-Bertani agar. Bacterial cell counts were determined after incubating the dishes at 37°C for 48 h. Bacterial culturing and sample handling were conducted in a sterile, clean chamber.
